# A Disintegrin and Metalloproteinase10 (ADAM10) Regulates NOTCH Signaling during Early Retinal Development

**DOI:** 10.1371/journal.pone.0156184

**Published:** 2016-05-25

**Authors:** Joseph A. Toonen, Adam Ronchetti, D. J. Sidjanin

**Affiliations:** 1 Department of Cell Biology, Neurobiology, and Anatomy, 8701 Watertown Plank Rd., Medical College of Wisconsin, Milwaukee, Wisconsin, United States of America; 2 Human and Molecular Genetics Center, 8701 Watertown Plank Rd., Medical College of Wisconsin, Milwaukee, Wisconsin, United States of America; Universidade Federal do ABC, BRAZIL

## Abstract

ADAM10 and ADAM17 are two closely related members of the ADAM (a disintegrin and metalloprotease) family of membrane-bound sheddases, which proteolytically cleave surface membrane proteins. Both ADAM10 and ADAM17 have been implicated in the proteolytic cleavage of NOTCH receptors and as such regulators of NOTCH signaling. During retinal development, NOTCH signaling facilitates retinal neurogenesis by maintaining progenitor cells in a proliferative state and by mediating retinal cell fates. However, the roles of ADAM10 and ADAM17 in the retina are not well defined. In this study, we set out to clarify the roles of ADAM10 and ADAM17 during early retinal development. The retinal phenotype of conditionally abated *Adam17* retinae (*Adam17* CKO) did not differ from the controls whereas conditionally ablated *Adam10* retinae (*Adam10* CKO) exhibited abnormal morphogenesis characterized by the formation of rosettes and a loss of retinal laminae phenotypically similar to morphological abnormalities identified in mice with retinal NOTCH signaling deficiency. Additionally, *Adam10* CKO retinae exhibited abnormal neurogenesis characterized by fewer proliferating progenitor cells and greater differentiation of early photoreceptors and retinal ganglion cells. Moreover, constitutive activation of the NOTCH1-intracellular domain (N1-ICD) rescued *Adam10* CKO abnormal neurogenesis, as well as abnormal retinal morphology by maintaining retinal cells in the progenitor state. Collectively these findings provide *in vivo* genetic evidence that ADAM10, and not ADAM17, is indispensable for proper retinal development as a regulator of NOTCH signaling.

## Introduction

During retinal development, all retinal cell types are derived from a single population of pluripotent retinal progenitor cells (RPCs). The birth order of retinal cells is unidirectional and highly conserved although at any given developmental time point there is an overlap in the generation of various retinal cell types [1–3]. In mice, retinal neurogenesis starts around E11 with the birth of ganglion cells followed by the birth of cone photoreceptors, horizontal and amacrine cells, with rod photoreceptors forming around birth and finally bipolar cells and Müller glia as the last retinal cell types born postnatally [1–3]. It has been proposed that RPCs undergo temporally regulated successive stages of competence to either generate a differentiated retinal cell type or to transit to the next stage of RPC competence that facilitates the birth of subsequent retinal cell types [1, 3].

NOTCH signaling is an evolutionarily conserved pathway involved in the development of most tissues. The role of NOTCH signaling is in the regulation of cell proliferation, cell death, cell fate determination, and differentiation [4, 5]. In mammals, there are four NOTCH receptors (NOTCH1-4) and five NOTCH ligands (JAG1, JAG2, DLL1, DLL3, DLL4) that exhibit both redundant and unique functions [4]. The canonical NOTCH pathway involves binding of a NOTCH ligand from the surface of adjacent cells to the NOTCH receptor thereby facilitating the subsequent NOTCH receptor cleavage at the S2 site followed by cleavage at the S3 and S4 sites resulting in the release of the NOTCH intracellular domain (NICD) from the cell membranes; once released the NICD translocates into the nucleus and forms a complex with RBPJ and MAML1 along with other cofactors to transcriptionally activate inhibitors of differentiation [6–8]. Therefore, one of the key roles of NOTCH signaling is maintaining progenitor cells in their undifferentiated state. Additionally, during retinal development NOTCH signaling facilitates neurogenesis by repressing retinal cell fates [9–15].

ADAM10 and ADAM17 are two closely related members of the ADAM family of proteins that proteolytically cleave or “shed” ectodomains of cell surface proteins [16, 17]. Both ADAM10 and ADAM17 have been implicated as sheddases of NOTCH receptors at the S2 cleavage site thereby facilitating subsequent cleavage at S3 and S4 sites by the γ-secretase complex [18–21]. In *C*. *elegans*, sup-17, the ortholog of ADAM10, and adm-4, the ortholog of ADAM17, are functionally redundant as NOTCH regulators [21]. In *Drosophila*, *Kuzbanian (kuz*), the fly ortholog of ADAM10, regulates NOTCH cleavage and signaling [18–20]. Phenotypically, *kuz* mutants exhibit neurogenic and ommatidial defects similar to those observed in the fly *Notch* mutants [18]. Mice deficient for *Adam10* die at E9.5 and phenocopy *Notch1* deficient mice [22] in contrast to *Adam17*^*-/-*^ mice that die at birth without phenotypic similarities to *Notch1* mouse mutants [23, 24]. Although findings from knock-out mice implicate ADAM10 in the proteolytic cleavage of NOTCH1, tissue culture studies have shown that ADAM17, and not ADAM10, cleaves NOTCH1 [25, 26]. Further studies determined that ADAM10 is indispensable for ligand-induced NOTCH1 signaling and ADAM17 mediates ligand-independent NOTCH1 cleavage [27, 28]. Therefore, it was proposed that different ADAMs might contribute to the NOTCH receptor cleavage in a tissue-specific manner with ADAM10 as the primary regulator of NOTCH1 cleavage *in vivo* [22]. Recent studies support this hypothesis showing that *in vivo* ADAM10 regulates NOTCH1 during brain [29], skin [30], intestinal [31], thymocyte [32], and vascular development [33].

During retinal development, the roles of ADAM10 and ADAM17 are unclear. Both ADAM10 and ADAM17 exhibit similar spatiotemporal expression in the developing chicken retina [34]. The early embryonic lethality of *Adam10*^*-/-*^ mice prior to the optic cup formation has precluded any evaluation of the role of ADAM10 in the retina [22]. However, pharmacological inhibition of ADAM10 partially rescued upregulated NOTCH1 signaling in mouse retinae deficient for *Srfp1* and *Srfp2* implicating ADAM10 as a regulator of NOTCH1 signaling during early retinal development [35]. By contrast, at birth *Adam17*^*-/-*^ mice do not exhibit any obvious retinal abnormalities suggesting that ADAM17 does not have an essential role during the early retinal development, although the perinatal death of *Adam17*^*-/-*^ mice precluded evaluations of the postnatal *Adam17* deficient mature retinae [23, 24].

The goal of this study was to determine the roles of ADAM10 and ADAM17 in the retina and establish whether ADAM10, ADAM17, or both regulate retinal NOTCH1 signaling. Our results demonstrate that ADAM10, and not ADAM17, is indispensable for early retinal development as a key regulator of NOTCH1 signaling.

## Materials and Methods

### Mice and clinical eye evaluation

All animal procedures were performed in accordance with NIH guidelines and approved by the Medical College of Wisconsin Institutional Animal Care and Use Committee (IACUC). Mice carrying a floxed *Adam10* allele (*Adam10*^*tm1*.*1Zhu*^) were generously provided by Tian Xu. Mice carrying a floxed *Adam17* allele (*Adam17*^*tm1*.*2Bbl*^), were generously provided by Carl Blobel. *Six3-Cre* mice carrying a *Cre* allele driven by the promoter of the *Six3* gene were generously provided by Yasuhide Furuta. *Gt(ROSA)26Sor* ^*tm1(NOTCH1)Dam*^/J. Each mouse strain was genotyped as previously described [24, 32, 36, 37] using primers in S1 Table. Mice with *Six3-Cre;Adam10*^*tm1Zh/tm1Zhu*^ or *Six3-Cre; Adam17*^*tm1Bbl/tm1Bbl*^ genotypes were referred to here as *Adam10* CKO mice and *Adam17* CKO mice respectively. Littermates with genotypes *Adam10*^*tm1Zhu/+*^ and *Adam10*^*tm1Zhu/ tm1Zhu*^ as well as *Adam17*^*tm1Bbl*/+^ and *Adam17*^*tm1Bbl/tm1Bbl*^ are referred to as controls.

Mouse eyes from the eight week old control, *Adam10* CKO, and *Adam17* CKO mice were examined with a Topcon SL-D8Z slit lamp biomicroscope with a Nikon SLR-based Photo Slit Lamp imaging system following mydriasis with 1% atropine sulfate (Bausch & Lomb) as previously described [38]. Control (n = 3) and *Adam17* CKO (n = 3) animals at 8 weeks of age were dark adapted for 2 hr and ERGs were recoded after a single flash of intensity sufficient to evoke the maximum retinal response as previously described [39].

### RT-PCR

Eyes from newborn mice (n = 3) of each genotype were collected and the cornea, iris, and lens were removed. RNA was isolated and reverse transcribed as previously described [40]. *Adam10 or Adam17* transcript levels were analyzed via semi-quantitative RT-PCR while in the exponential phase of the PCR amplification using *Gapdh* as an internal control as previously described [40] using primers in S1 Table. PCR band intensities were quantified using ImageJ software (http://rsbweb.nih.gov/ij/) and are expressed relative to *Gapdh*. The results represent at least three independent experiments performed in triplicates and the data is expressed as mean ± SEM. Comparison between the genotype groups was analyzed by Student’s *t*-test and *P*<0.05 was considered as statistically significant.

### Histology and immunohistochemistry

Tissues were fixed in 4% PFA, or Davidson’s solution, embedded in paraffin, sectioned to 4 μm thickness and Hematoxylin and Eosin (H&E) stained as previously described [38]. For immunohistochemistry, following deparaffinization and rehydration, antigen retrieval was performed using 1x citrate buffer pH 6.0 (Life Technologies) as previously described [38] and then sections were blocked in 10% normal serum with 1% BSA in 1xPBS for 1–2 hr and incubated with primary antibody in 1xPBS with 1% BSA O/N at 4°C. 2x 5 min washes in 1xPBS, 0.025% Triton X-100 were followed by secondary antibody incubation in 1xPBS with 1% BSA for 1 hr at RT. Primary antibodies used in this study include: ADAM10 (R&D Systems), BRN3B, ADAM17 OTX2, and N-cadherin (Abcam), ISL1 (Hybridoma Bank), β-catenin, NOTCH1 and HES1 (Cell Signaling). Secondary antibodies used were Alexa Fluor 546 goat-anti rabbit and Alexa Fluor 488 goat-anti mouse (Life Technologies). All slides were counterstained with DAPI (Life Technologies), mounted using Prolong gold anti-fade reagent (Life Technologies) mounting media, and photographed with a Nikon DS-Fi1 camera on a Nikon eclipse 80i microscope. The percentage of OTX2 and ISL1positive cells were determined by counting the numbers of OTX2 and ISL1 positive cells and total DAPI positive cells from a minimum of 3 separate genotypes with at least 10 sections per genotype. Significance was calculated via Students *t*-test (GraphPad Prism), and *P*<0.05 was considered significant.

### EdU, BrdU, and TUNEL assays

EdU (Life Technologies) or BrdU was injected intraperitoneally at a dose of 125 mg/kg of body weight into pregnant dams, 13.5 days after a vaginal plug was found and sacrificed 2 hr later. Embryos were fixed in 4% PFA for 2 hours and dehydrated through a graded series of ethanol washes, were paraffin embedded, and cut into 5 μm sections. EdU detection was performed with Click-iT EdU Alexa Fluor 488 Imaging Kit (Life Technologies) according to the manufacturer’s protocol and BrdU was detected using a mouse monoclonal anti-BrdU antibody conjugated to Alexa Flour 488 (Life Technologies). TUNEL staining was performed utilizing the ApopTag Plus In Situ Apoptosis Fluorescein Detection Kit (Millipore) according to the manufacturer’s recommendations. DAPI (Life Technologies) was used to stain the DNA, slides were mounted using Prolong gold anti-fade reagent (Life Technologies) and imaged using a Nikon DS-Fi1 camera on a Nikon Eclipse 80i microscope. The percentage of EdU, BrdU, and TUNEL positive cells after counting the numbers of EdU, BrdU or TUNEL positive cells and total DAPI positive cells from a minimum of 3 separate genotypes with at least 10 sections per genotype. Significance was calculated via a *t*-test (GraphPad Prism) and *P*<0.05 was considered significant.

## Results

### ADAM10 and ADAM17 are ubiquitously expressed retinal proteins

**Fig 1:**
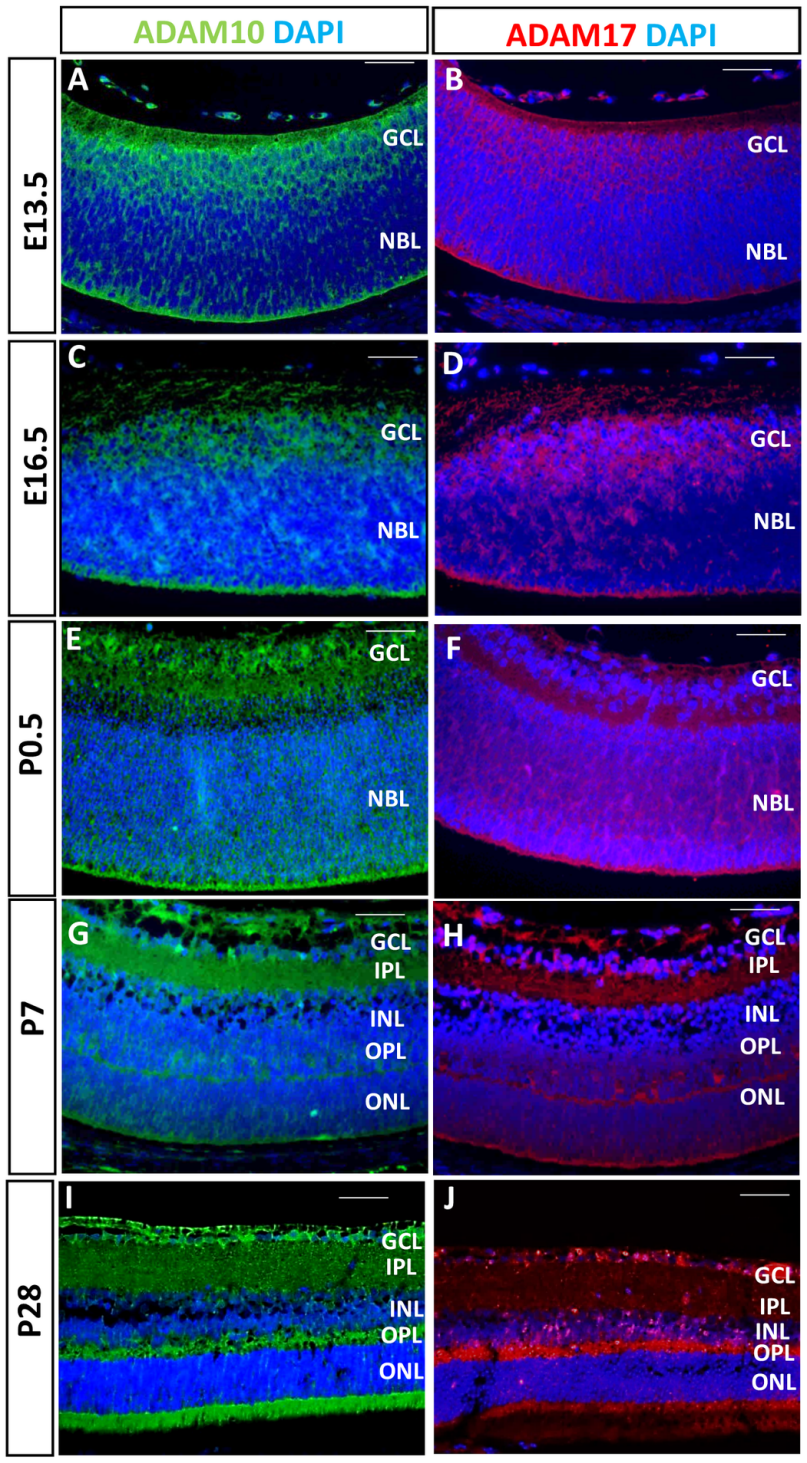
Expression of ADAM10 and ADAM17 in the retina. Immunostaining of control E13.5 retinae for ADAM10 (A) and ADAM17 (B), E16.5 retinae for ADAM10 (C) and ADAM17 (D) and P0.5 retinae for ADAM10 (E) and ADAM17 (F) revealed similar expression patterns in the ganglion cell layer (GCL) and neuroblastic layer (NBL). Immunostaining of P7 retinae for ADAM10 (G) and ADAM17 (H) and P28 retinae for ADAM10 (I) and ADAM17 (J) also revealed similar expression patterns in the inner plexiform layer (IPL), outer plexiform layer (OPL) and in the photoreceptors. INL = inner nuclear layer; ONL = outer nuclear layer. Nuclei were stained with DAPI. Scale bars = 50 μm.

As the initial step, we evaluated the expression of ADAM10 and ADAM17 during retinal development in mice. In control embryonic E13.5, E16.5 and newborn P0.5 retinae both ADAM10 (Fig 1A, 1C and 1E) and ADAM17 (Fig 1B, 1D and 1F) exhibit ubiquitous expression in ganglion cell and neuroblastic layers. By P7, as the retina starts to mature, ADAM10 and ADAM17 are expressed in the ganglion cell layer, inner and outer plexiform layers, and in the photoreceptors (Fig 1G and 1H) and this pattern of expression is maintained in the adult retina for both ADAM10 (Fig 1I) and ADAM17 (Fig 1J).

### ADAM10, but not ADAM17, is required for early retinal development

**Fig 2:**
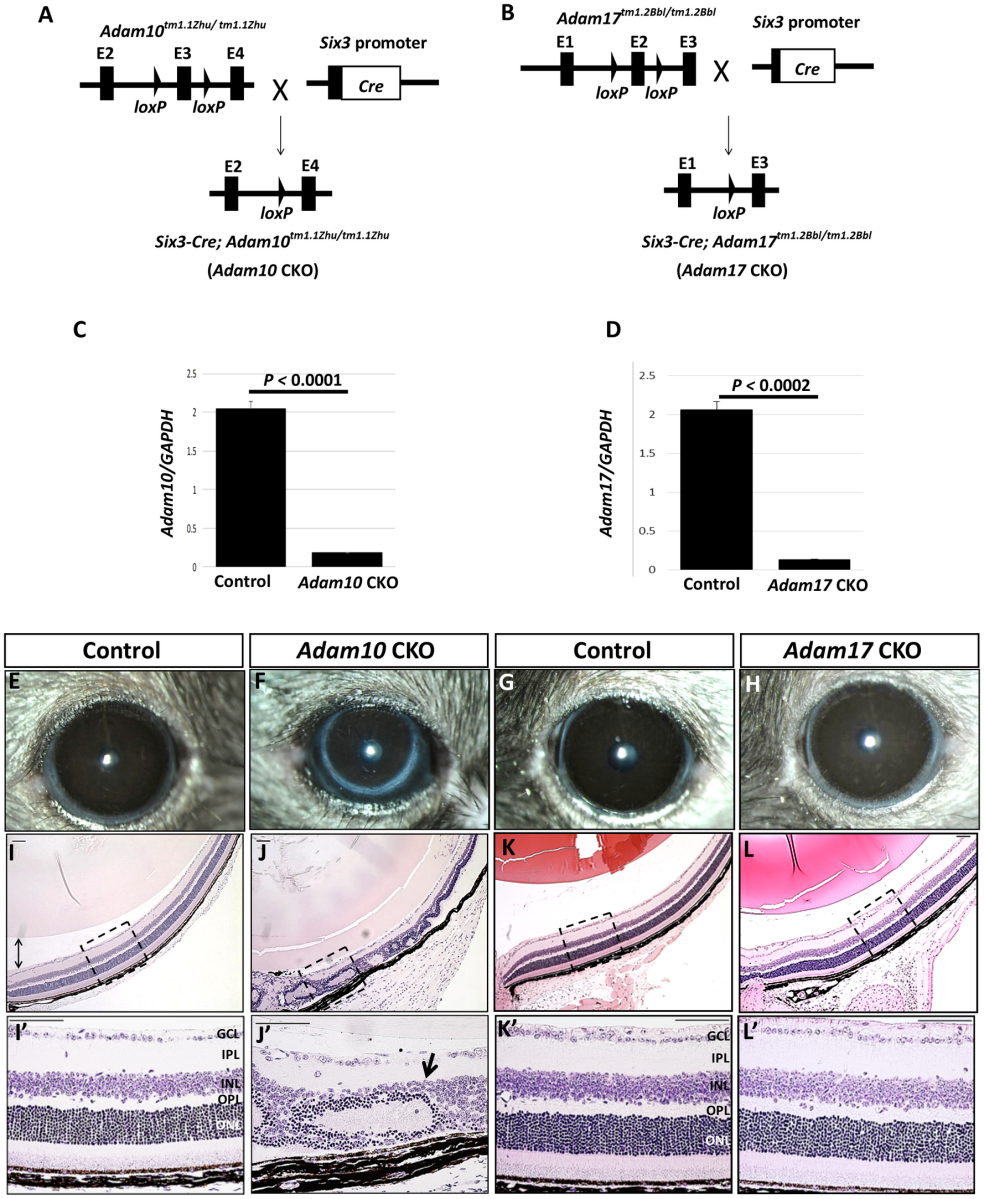
Conditional ablation of *Adam10* and *Adam17* in the retina. (A) Mice carrying homozygous floxed *Adam10* allele (*Adam10*^*tm1*.*1Zhu*^) were crossed with *Six3*-*Cre* transgenic mice expressing *Cre* in the optic cup and optic stalk resulting in conditionally ablated *Adam10* in the retina. (B) A similar approach was taken to generate *Adam17 CKO* mice after homozygote *Adam17*^*tm1*.*2Bbl*^ mice were crossed to *Six3-Cre* mice. (C) Semi-quantitative analysis of the relative expression of *Adam10* transcript containing exon 3 normalized to *Gapdh* expression from RNA isolated from newborn control and *Adam10* CKO littermates revealed significantly reduced (*P<*0.0001; n = 3) levels in *Adam10* CKO retinae when compared to the controls. (D) Semi-quantitative analysis of the relative expression of exon 2 containing *Adam17* mRNA normalized to *Gapdh* expression from RNA isolated from newborn control and *Adam17* CKO retinae also revealed significantly reduced (P*<*0.0002; n = 3) levels in *Adam17* CKO retinae when compared to the controls. In both (C) and (D), the bars represent mean values ± SEM. Significance was established following Student’s *t-*test analysis and *P*<0.05 was considered significant. (F) *Adam10* CKO eyes appeared smaller when compared to the eyes in the control mice (E) whereas *Adam17* CKO eyes (H) that did not appear to differ from age-matched control littermates (G). H&E staining of *Adam10* CKO eyes at 8 weeks of age (J) revealed severe retinal abnormalities characterized by the formation of rosettes (J’, arrow) in contrast to the retinal laminae in the age-matched controls (I-I’). Histological analysis of *Adam17* CKO eyes (L-L’) did not identify any morphological differences when compared to the age-matched controls (K-K’). Figures shown in (I’) through (L’) are enlarged images of the areas depicted by the dashed boxes in Figures (I) through (L). GCL = ganglion cell layer, IPL = inner plexiform layer, INL = inner nuclear layer, OPL = outer plexiform layer, ONL = outer nuclear layer. Scale bars = 50 μm.

To determine the roles of ADAM10 and ADAM17 during early retinal development, we generated mice with retina-specific ablation of *Adam10* (*Adam10* CKO), as well as retina-specific ablation of *Adam17* (*Adam17* CKO) (Fig 2A and 2B). In newborn *Adam10* CKO and *Adam17* CKO retinae, only residual levels of *Adam10* transcript containing exon 3 (Fig 2C) and *Adam17* transcript containing exon 2 (Fig 2D) were identified when compared to the controls. Therefore, *Adam10* and *Adam17* were successfully disrupted in most retinal cells in *Adam10* CKO and *Adam17* CKO mice respectively.

The evaluation of adult (8 weeks of age) *Adam10* CKO eyes by observation revealed a smaller eye size (Fig 2F) when compared to age-matched control littermates (Fig 2E). *Adam17* CKO mice did not exhibit any obvious eye size differences (Fig 2H) when compared to age-matched control littermates (Fig 2G). Histological analysis of adult *Adam10* CKO eyes identified profoundly disrupted retinal laminae (Fig 2J and S1B Fig) characterized by the formation of rosettes (Fig 2J’). Histological analysis of *Adam17* CKO eyes and retinae (Fig 2L and 2L’ and S1D Fig) did not identify any morphological differences when compared to age-matched control littermates (Fig 2K and 2K’ and S1C Fig). To exclude the possibility that *Adam17* CKO retinae may exhibit functional, but not morphological abnormalities we performed electroretinograms (ERGs). Our analysis did not identify any differences in ERGs between the control and *Adam17* CKO mice (not shown). Collectively these findings provided evidence that ADAM10, and not ADAM17, is required for early retinal development.

### The onset and progression of retinal phenotypes in *Adam10* CKO mice

**Fig 3:**
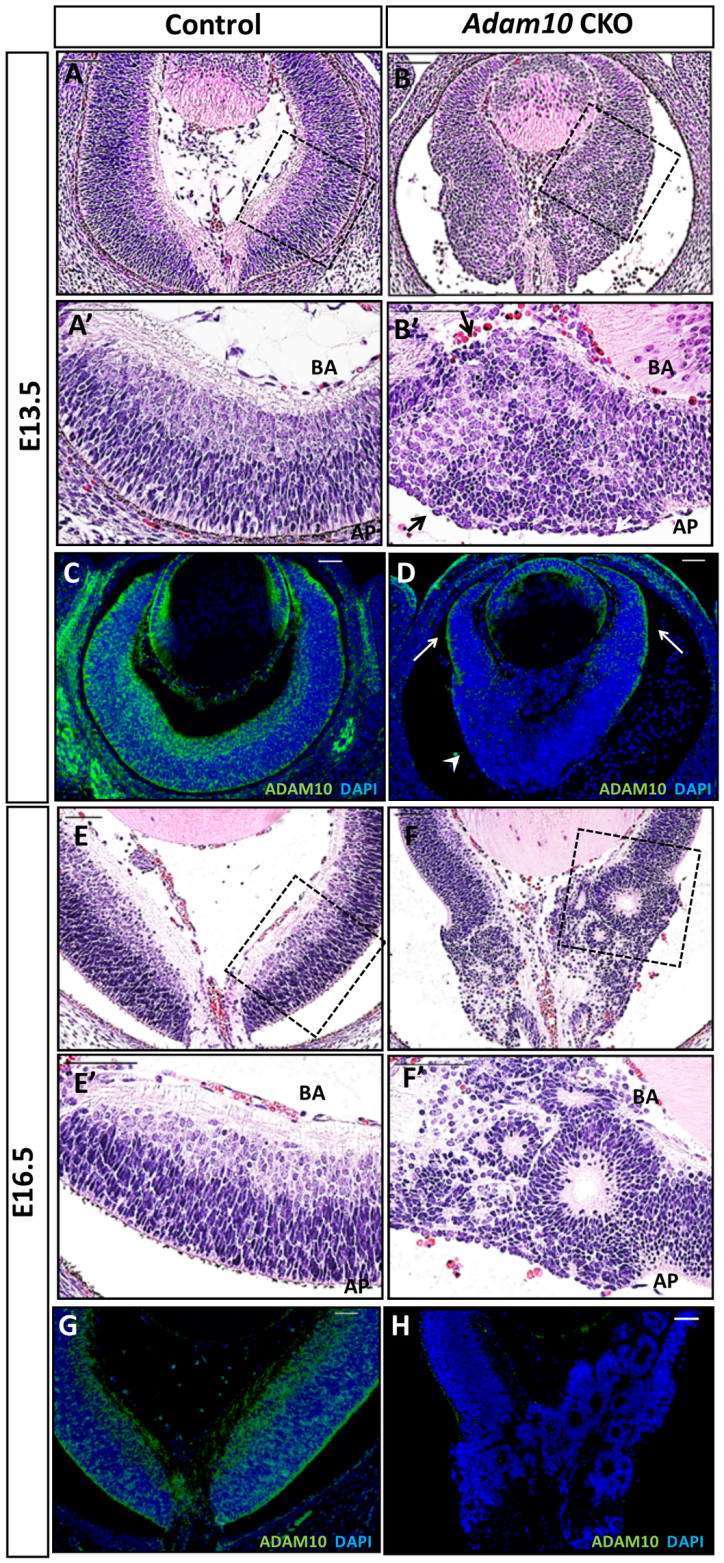
Retinal morphology in control and *Adam10* CKO mice. H&E staining revealed first abnormalities in E13.5 *Adam10* CKO retinae (B) within the central region characterized by rounded and highly disorganized nuclei along the apical-basal axis resulting in disrupted basal and apical surfaces (B’, arrows) in contrast to the highly organized control retinae (A-A’). ADAM10 immunostaining of E13.5 *Adam10* CKO retinae (D, arrowhead) revealed an absence of ADAM10 expressions in most cells within the central retina when compared to the age-matched controls (C). However, residual ADAM10 expression was detected in the peripheral retinae of *Adam10* CKO mice (D, arrows). H&E staining of E16.5 *Adam10* CKO retinae (F) identified within the central region rosettes of various sizes (F’) in contrast to the highly organized retinal laminae in the age-matched control retinae (E-E’). Immunostaining of E16.5 *Adam10* CKO retinae (H) did not identify any ADAM10-positive cells in contrast to the age-matched controls (G). BA = basal surface, AP = apical surface. Scale bars = 50 μm.

The first morphological abnormalities in *Adam10* CKO retinae were evident at E13.5 characterized by disrupted cellular architecture (Fig 3B). Along the apical-basal axis nuclei from *Adam10* CKO retinae appeared rounded and disorganized resulting in misshapen apical and basal surfaces (Fig 3B’). By contrast, the E13.5 control retinae exhibited elongated and organized nuclei with distinct apical and basal surfaces (Fig 3A and 3A’). While morphological abnormalities were identified within the *Adam10* CKO central retinae some regions of peripheral retinae retained undisrupted retinal cell organization (Fig 3B). It was reported previously that *Six3-Cre* is expressed across the entire retina although the highest expression is within the central retina [36]. To explore this further, we immunostained the control and the *Adam10* CKO retinae for ADAM10. Within the central *Adam10* CKO retinae, the majority of cells did not express ADAM10 (Fig 3D and S2B Fig). However, at the retinal periphery, we identified residual ADAM10 immunostaining (Fig 3D and S2B Fig). This finding suggested that milder retinal abnormalities identified at the retinal periphery of E13.5 *Adam10* CKO may have been caused by the lower efficiency of CRE-mediated *Adam10* ablation relative to the central retina. By E16.5 the *Adam10* CKO retinae started to exhibit rosettes within the central retinae (Fig 3F); the identified rosettes were of different sizes (Fig 3F’). Immunostaining of E16.5 *Adam10* CKO retinae for ADAM10 did not identify any residual staining (Fig 3H). We also morphologically evaluated the postnatal retinal development of *Adam10* CKO mice. By P0.5 in *Adam10* CKO retinae, the formation of rosettes expanded further towards the retinal periphery (S2D and S2D’ Fig) and by P7 the rosettes were present throughout the entire retina (S2F and S2F’ Fig).

### ADAM10 inactivation disrupts retinal neurogenesis

**Fig 4:**
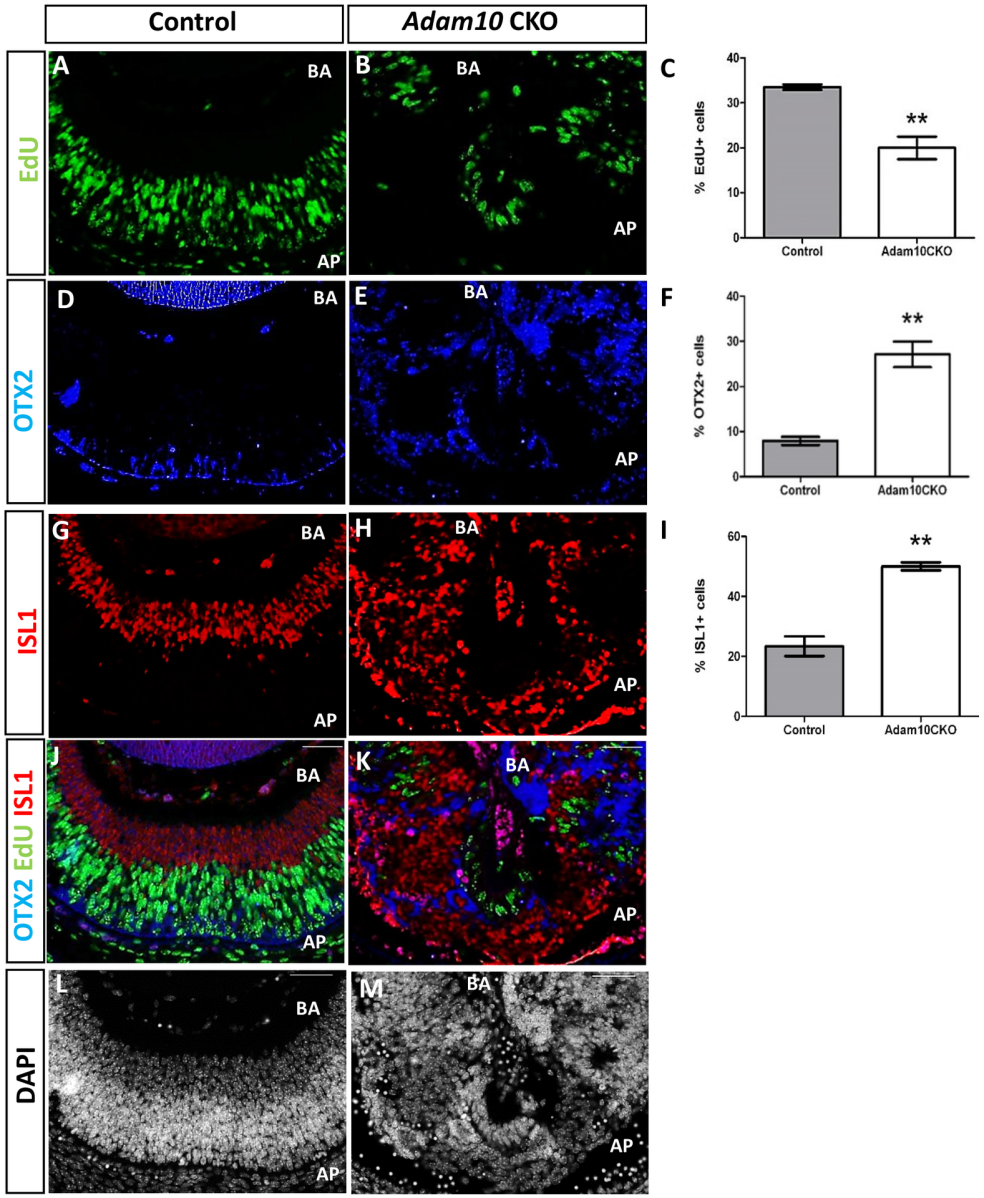
Retinal neurogenesis at E13.5 in control and *Adam10* CKO mice. In the control retinae (A) the EdU-positive cells were highly organized along the apical surface with no EdU-positive cells present at the basal surface. By contrast, in the *Adam10* CKO eyes (B) within the central retinae a few highly disorganized EdU-positive cells were identified. Quantification analysis (C) revealed in control retinae 33.50 ± 0.61% (gray bar) and in *Adam10* CKO retinae 20.01± 2.5% (white bar) EdU-positive cells indicating a significantly lower (**P = 0.019; n = 3) percentage of EdU-positive cells in the *Adam10* CKO retinae. In the control retinae (D), sparse OTX2-positive immunostaining was identified in cells along the apical surface. In *Adam10* CKO retinae (E), OTX2-positive cells were present in all retinal layers. Quantification analysis (F) revealed in control retinae 7.95 ± 0.9% (gray bar) and in *Adam10* CKO retinae 27.1 ± 2.8% (white bar) OTX2- positive cells indicating a significantly greater (**P = 0.003; n = 3) percentage of OTX2-positive cells in *Adam10* CKO retinae. In the control retinae (G), ISL1-positive cells were identified within the basal layer whereas in *Adam*10 CKO retinae (H) ISL1-positive cells were highly disorganized and present in all retinal layers. Quantification analysis (I) revealed in the control retinae 23.4 ± 3.2% (gray bar) and in *Adam10* CKO retinae 50.1 ± 1.3% (white bar) ISL1-positive cells indicating a significantly greater (**P = 0.004; n = 3) percentage of ISL1-positive cells in the *Adam10* CKO retinae. In both the control (J) and *Adam10 CKO* retinae (K), the OTX2-positive and ISL1-positive cells did not co-stain for EdU. DAPI staining of the control and *Adam10* CKO retinae are shown in (L) and (M) respectively. Bars in (C), (F), and (I) represent mean values ± SEM. AP = apical surface. BA = basal surface. Scale bar = 50 μm.

Next, we wanted to determine if *Adam10* CKO retinae exhibited disrupted neurogenesis. At E13.5 in control retinae, EdU-positive cells were highly organized and restricted to the neuroblastic layer (Fig 4A) whereas in E13.5 *Adam10* CKO retinae EdU-positive cells were highly disorganized (Fig 4B). Quantification analysis revealed a significantly lower (*P =* 0.019; n = 3) percentage of EdU-positive cells in the *Adam10* CKO retinae when compared to the controls (Fig 4C). Next, we hypothesized that the fewer proliferating cells identified in *Adam10* CKO retinae (Fig 4A–4C) may be caused by precocious retinal cell differentiation. Immunostaining of the control retinae for orthodenticle homeobox 2 (OTX2), which is a marker for early photoreceptors [41], revealed sparse OTX2-positive staining in cells along the retinal apical surface consistent with the development of early photoreceptors (Fig 4D). By contrast, *Adam10* CKO retinae exhibited highly disorganized OTX2-positive cells (Fig 4E). Quantification analysis revealed a significantly greater (*P =* 0.003; n = 3) percentage of OTX2-positive cells in the *Adam10* CKO retinae when compared to the controls (Fig 4F). We also evaluated the ISL1 expression as a marker for early ganglion cells [42]. In control retinae, ISL1 immunostaining was restricted to the cells at the retinal basal layer consistent with the developing ganglion cells (Fig 4G). In the *Adam10* CKO retinae, ISL1-positive cells were highly disorganized (Fig 4H). Quantification analysis revealed a significantly greater (*P =* 0.004; n = 3) percentage of ISL1-positive cells in E13.5 *Adam10* CKO when compared to the control retinae (Fig 4I). Similar findings were observed following immunostaining with BRN3B (S3A–S3C Fig), a marker for early ganglion cells [42]. In both the control (Fig 4J) and *Adam10* CKO retinae (Fig 4K), the OTX2-positive and ISL1-positive cells did not co-stain for EdU, indicating that OTX2-positive and ISL1-positive cells had exited the cell cycle.

### Characterization of rosettes in E16.5 *Adam10* CKO retinae

**Fig 5:**
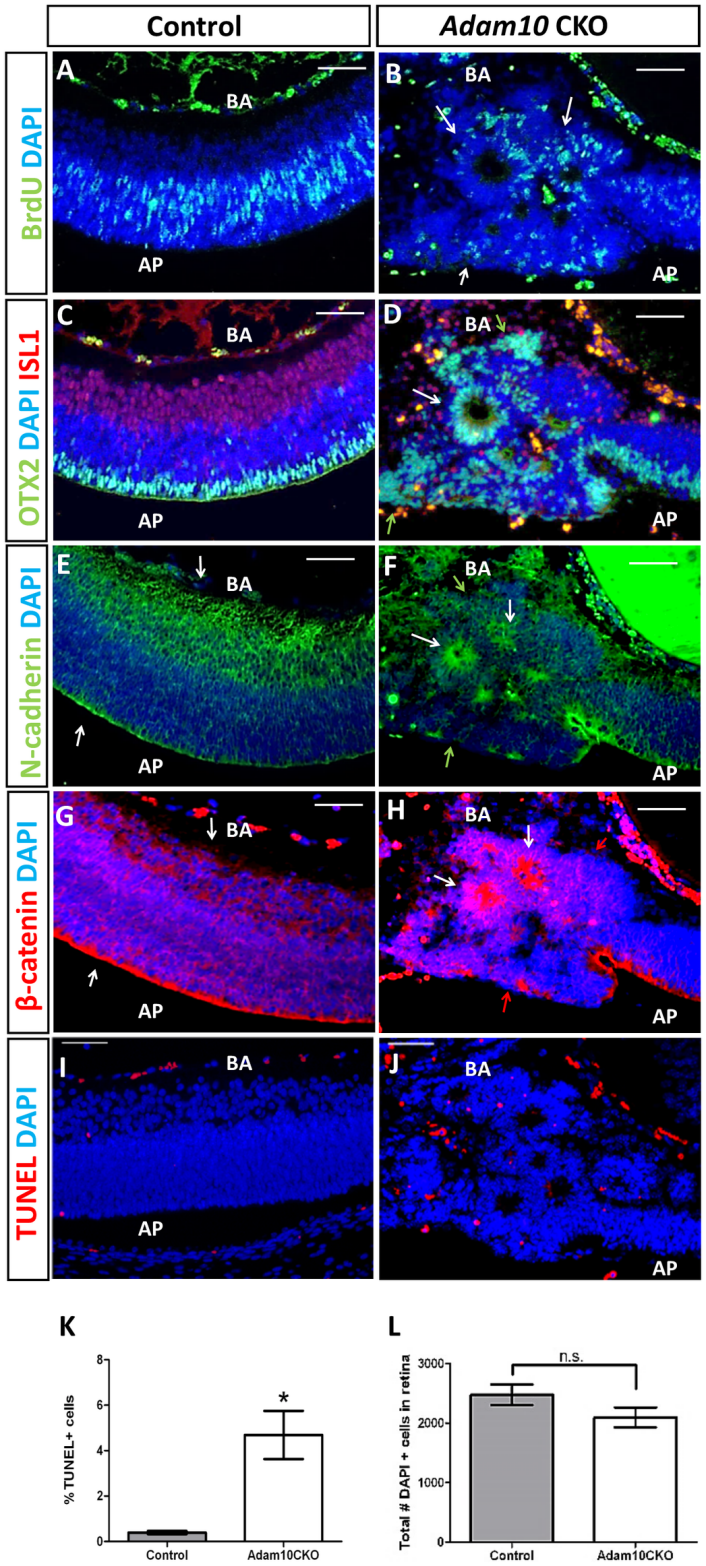
Characterization of rosettes in E16.5 *Adam10* CKO retinae. In controls (A) BrdU-positive cells were identified in the neuroblastic layer. In *Adam10* CKO retinae, BrdU-positive cells were identified in the outer cell layer of rosettes (B, arrows). In controls (C) immunostaining for OTX2 (green) and ISL1 (red) marked early photoreceptor cells at the apical surface and ganglion cells at the basal surface. In *Adam10* CKO retinae (D) the OTX2-positive cells (D, green) formed rosette lumens (D, white arrow), although OTX2-positive cells were also present at the apical and basal surfaces (green arrows in D). ISL1-positive cells (D, red) did not associate with rosettes and were scattered through the entire retina. In control retinae, N-cadherin (E) and β-catenin (G) were expressed in all retinal cells although the highest expression was in the cells forming the apical surface (white arrows in E and G). In *Adam10* CKO retinae cells forming the rosette lumens were highly expressing N-cadherin and β-catenin (white arrows F and H); cells at the apical and basal surface were intermittently expressing N-cadherin (green arrows in F) and β-catenin (red arrows in H). Very few TUNEL-positive cells were identified in the control (I) and *Adam10* CKO (J) retinae. Quantification analysis (K) identified in the controls 0.4 ± 0.01% (gray bar) and in *Adam10* CKO 4.69 ± 0.11% (white bar) TUNEL-positive cells indicated a significantly greater (*P = 0.003; n = 3) percentage of TUNEL-positive cells in the *Adam10* CKO retinae. Quantification analysis (L) identified 2476 ± 173 (gray bar) DAPI-positive cells in controls and 2096 ± 167 (white bar) DAPI-positive cells in *Adam10* CKO retinae indicating that the overall number of retinal cells did not significantly differ (*P* = 0.19; n = 3) between the two genotypes. Bars in (K) and (L) represent mean values ± SEM. Significance was established following analysis with Student’s *t-*test and *P<*0.05 was considered significant. In all panels, nuclei were stained with DAPI. AP = apical surface. BA = basal surface. n.s. = not significant. Scale bar = 50 μm.

Morphological evaluation of *Adam10* CKO retinae revealed the formation of rosettes at E16.5 (Fig 3F and 3F’). Next we set to characterize which cells formed the rosettes in E16.5 *Adam10* CKO retinae. In the control, the retinae BrdU-positive cells are positioned between the OTX2-positive photoreceptors on the apical side and ISL1-positive ganglion cells at the basal side (Fig 5A and 5C). Immunostaining of *Adam10* CKO retinae revealed that BrdU-positive cells were forming the rosette outer cellular layers (Fig 5B) whereas OTX2 positive photoreceptors were forming the rosette inner cellular layers (Fig 5D). However, in the *Adam10* CKO retinae, OTX2-positive photoreceptors were also identified as highly disorganized cells present at the apical and basal surface as well as cells randomly scattered throughout the retina (Fig 5D). The ISL1-positive cells did not associate with rosettes and appeared to be randomly distributed throughout the *Adam10* CKO (Fig 5D). It was shown previously that both N-cadherin and β-catenin are indispensable for the formation of retinal laminae [43, 44], thus we wanted to determine if N-cadherin and β-catenin expression had been altered in the *Adam10* CKO retinae. In the control retinae both N-cadherin and β-catenin (Fig 5E–5G) were expressed throughout the retina especially in the cells forming apical and basal surfaces consistent with previous reports [44]. In the *Adam10* CKO retinae both N-cadherin (Fig 5F) and β-catenin (Fig 5H) were highly expressed in cells forming the rosette lumens whereas cells forming apical and basal surfaces intermittently expressed both N-cadherin and β-catenin. Next, we wanted to determine if cell death was contributing to the formation of rosettes. In E16.5 control retinae, we identified a very few TUNEL-positive cells (Fig 5I) whereas in *Adam10* CKO the TUNEL-positive cells were scattered throughout all layers of the retinae (Fig 5J). Although a significantly greater (*P =* 0.003; n = 3) percentage of TUNEL-positive cells were identified in *Adam10* CKO retinae (Fig 5H) when compared to controls, the overall percentage of TUNEL-positive cells was very small (<5%) in both the WT and *Adam10* CKO retinae (Fig 5K). Consistent with this, the overall number of DAPI positive cells did not significantly differ (*P =* 0.19; n = 3) between the two genotypes (Fig 5L).

### ADAM10 regulates NOTCH signaling

**Fig 6:**
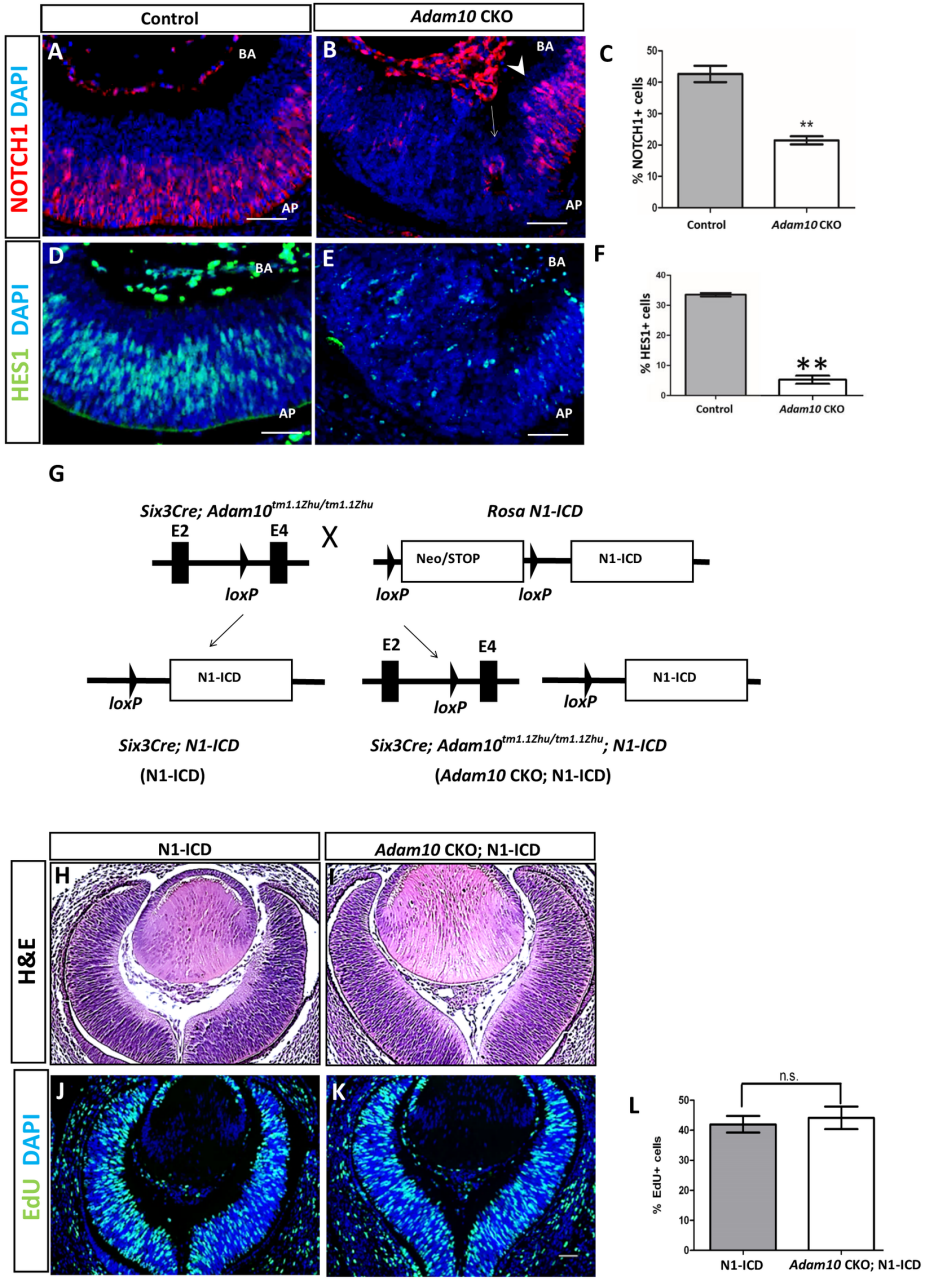
Overexpression of N1-ICD in *Adam10* CKO retinae rescues aberrant retinal morphogenesis and neurogenesis. In controls (A) NOTCH1-positive cells were restricted to the neuroblastic layer. In *Adam10* CKO retinae, within the central retinae only a very few NOTCH1-positive cells were identified associated with rosettes (B, arrow) although towards the retinal periphery NOTCH1-positive cells were identified restricted to the neuroblastic layer (B, arrowhead). Quantification analysis (C) identified in controls 42 ± 1.2% (gray bar) and in *Adam10* CKO 22.79 ± 0.11% (white bar) NOTCH1-positive cells indicating a significantly fewer (**P = 0.001; n = 3) percentage of NOTCH1-positive cells in *Adam10* CKO retinae when compared to the controls. In controls HES1, immunostaining was restricted to the neuroblastic layer (D) whereas in *Adam10* CKO retinae a very few HES1-positive cells were identified within the central retinae and HES1-positve cells appeared disorganized (E). Quantification analysis (F) identified in controls 34 ± 0.01% (gray bar) and in *Adam10* CKO retinae 4.7 ± 0.2% (white bar) HES1-positive cells indicating a significantly fewer (**P = 0.0004; n = 3) percentage of HES1-positive cells in *Adam10* CKO retinae when compared to controls. (G) *Adam10* CKO mice were crossed to *Rosa*^*N1-IC*^ (N1-ICD) mice harboring a Neo/STOP cassette flanked by *loxP* sites upstream of a sequence encoding the intracellular domain of the *NOTCH1* gene thereby generating *Adam10* CKO; N1-ICD mice that exhibit constitutive expression of N1-ICD in the same retinal cells in which *Six3-*CRE led to ablation of *Adam10*. H&E staining revealed in N1-ICD mice (H) and *Adam10* CKO;N1-IC mice (I) highly organized retinae. In N1-ICD retinae (J) and *Adam10* CKO;N1-IC retinae (K) EdU-positive cells were present in all retinal layers. Quantification analysis (L) revealed in N1-ICD retinae 41.99 ± 2.78% (gray bar) and in *Adam10* CKO retinae 44.15 ± 3.76% (white bar) EdU-positive cells indicating that the percentage of EdU positive retinal cells did not significantly differ (*P =* 0.24; n = 3). Bars in (C), (F), and (L) represent mean values ± SEM. Significance was established with the Student’s *t-*test and *P<*0.05 was considered significant. AP = apical surface. BA = basal surface. Scale bars = 50 μm.

Next, we set out to determine if disrupted NOTCH1 signaling contributed to the retinal phenotypes identified in *Adam10* CKO retinae. Immunostaining of E13.5 control retinae revealed that NOTCH1 positive immunostaining was restricted to the neuroblastic layer (Fig 6A). By contrast in E13.5 *Adam10* CKO retinae very few cells within the central retina stained positive for NOTCH1, although towards the retinal periphery highly disorganized NOTCH1 positive cells were evident (Fig 6B). Quantification analysis confirmed a significantly (*P =* 0.001; n = 3) lower percentage of NOTCH1 positive cells in E13.5 *Adam10* CKO retinae when compared to the age-matched controls (Fig 6C). Immunostaining for HES1, which is a NOTCH1 downstream target [15], revealed a very few HES1-immunopositive cells within the central E13.5 *Adam10* CKO retinae that appeared highly disorganized (Fig 6D). However, at the retinal periphery HES1 positive cells were identified restricted to the neuroblastic layer (not shown). Quantification analysis confirmed a significantly (*P =* 0.0004; n = 3) lower percentage of HES1-positive in E13.5 *Adam10* CKO retinae when compared to the age-matched controls.

In order to determine if disrupted NOTCH1 signaling is directly caused by a functional loss of ADAM10, we utilized a genetic approach. We focused on establishing if overexpression of the NOTCH1-intracellular domain (N1-ICD) can rescue aberrant morphogenesis and/or neurogenesis identified in *Adam10* CKO retinae. We crossed *Adam10* CKO mice to *Gt(ROSA)26Sor* ^*tm1(NOTCH1)Dam*^/J mice and generated mice with genotypes *Six3-Cre;Adam10* CKO;*Rosa*^*N1-IC/+*^ (*Adam10* CKO;N1-ICD) wherein the same retinal cells *Six3-*driven CRE recombinase activity deleted *Adam10* exon 3 and overexpressed N1-ICD; mice with *Six3-Cre;Rosa*^*N1-IC/+*^ (N1-ICD) genotypes, where *Six3*-CRE-mediated constitutive retinal activation of N1ICD, were used as controls (Fig 6G). At E13.5 cells, both N1-ICD retinae (Fig 6H) and *Adam10* CKO;N1-ICD retinae (Fig 6I), were highly organized along the apical-basal axis with well-defined apical and basal surfaces. EdU incorporation revealed that in both N1-ICD retinae (Fig 6J) and *Adam10* CKO; N1-ICD retinae (Fig 6K) proliferative cells were present in all retinal layers suggesting that all retinal cells in these mice were maintained in the progenitor state. The percentage of EdU positive cells did not significantly differ between the genotypes (Fig 6L). To explore this further, we immunostained E13.5 N1-ICD retinae and *Adam10* CKO; N1-ICD retinae for OTX2 and ISL1 as early photoreceptor and ganglion cell markers respectively. Within the central region of both N1-ICD retinae (S4A Fig) and *Adam10* CKO; N1-ICD (S4B Fig) we did not identify any OTX2-positive or ISL1-positive cells. However, at the retinal periphery very few OTX2-positive (<1%) and ISL1-positive cells (<3%) were identified in both N1-ICD (S4C Fig) and *Adam10* CKO; N1-ICD retinae (S4D Fig). Quantification analysis did not identify a significant difference between N1-ICD and *Adam10* CKO;N1-ICD retinae in the percentage of OTX2-positive and ISL1-positive cells (S4E and S4F Fig).

## Discussion

In this study, we show that ADAM10 is indispensable for retinal neurogenesis as well as the formation of retinal laminae. The rescue of aberrant retinal phenotypes in ADAM10-deficient retinae by overexpression of the N1-ICD provides *in vivo* genetic evidence that ADAM10 is a key regulator of NOTCH1 signaling. In mice during early retinal development NOTCH1 maintains retinal cells in the undifferentiated progenitor state, but it also has a role in repressing photoreceptor cell fate [10, 11]. As such the role of NOTCH1 signaling has been proposed to have a key role in assuring the sufficient number of progenitor cells necessary for the development of all retinal cell types [10, 11]. Results from this study showed that ADAM10 deficiency decreased the pool of retinal progenitor cells and increased the production of early photoreceptors further supporting the idea that ADAM10 is a regulator of NOTCH1 signaling. However, our results also show that ADAM10 retinal deficiency increased the production of ganglion cells, a phenotype not identified in mice with NOTCH1-deficient retinae [10, 11]. ADAM10 ablation in the developing retina phenocopies aberrant retinal neurogenesis in mice with retinae deficient for both NOTCH1 and NOTCH3 [45] as well as in mice with retinae deficient for RPBJ [12]. While it is well established that NOTCH1 is a regulator of the progenitor cell pool and represses photoreceptor fate, recent studies have shown both NOTCH3 and NOTCH1 contribute to the repression of ganglion cell fate [45]. Furthermore both NOTCH1 and NOTCH3 signal through RBPJ as a common downstream target [12]. Therefore, it was hypothesized that NOTCH1+NOTCH3>RBPJ repress retinal ganglion cell fate. We propose that ADAM10 is a common NOTCH signaling regulator by facilitating the cleavage of both NOTCH1 and NOTCH3 which in turn facilitates maintenance of the progenitor cell pool and repression of both photoreceptors and ganglion cells. In support of this hypothesis are recent reports showing that in cell culture ADAM10 cleaves NOTCH3 via a similar mechanism as NOTCH1 [46]. Additionally, during retinal development expression of *NOTCH1* and *NOTCH3* spatially and temporally coincide [47]. However, further studies are needed to unequivocally establish if ADAM10 mediates the cleavage of both NOTCH1 and NOTCH3 during early retinal neurogenesis.

In addition to aberrant neurogenesis, ADAM10 retinal ablation also results in severely disrupted formation of retinal laminae. Prior to differentiation, RPCs are positioned along the apical-basal axis and are attached to both the apical and basal surfaces [48, 49]. As RPCs start to differentiate, they undergo detachment allowing the cells to migrate to the appropriate retinal layer thereby establishing the retinal laminae and neuronal circuitry required for vision [48, 49]. Our results show that ADAM10 retinal deficiency results in the formation of rosettes similar to the rosettes reported for mice with disrupted retinal NOTCH1 signaling [9–15]. It was shown that RBPJ retinal deficiency causes discontinuous expression of β-catenin resulting in the formation of rosettes; ectopic overexpression of β-catenin in RBPJ-deficient retinae rescued the rosette formation without rescuing neurogenesis defects [13]. How disrupted retinal NOTCH1 signaling alters β-catenin expression still remains unclear. Active β-catenin is a downstream effector of Wnt signaling [50], but active β-catenin is also bound to N-cadherin at the cell membranes [51, 52]. A functional deficiency of either N-cadherin or β-catenin disrupts the formation of retinal laminae without disrupted neurogenesis [43, 44, 53, 54]. However, it was shown previously that ADAM10 mediates the cleavage of N-cadherin and as such regulates the release of active β-catenin into the cytoplasmic pool [51]. At this point, it is unclear if the rosetting phenotypes observed in ADAM10-deficient retinae are solely caused by the disrupted NOTCH1 signaling or if disrupted ADAM10-mediated cleavage of N-cadherin also contributes to the aberrant retinal morphogenesis. The rescue of aberrant retinal morphology in ADAM10-deficeint retinae by overexpression of N1-ICD supports the idea that the failure in the formation of laminae in the ADAM10-deficeint retinae is solely caused by disrupted NOTCH1 signaling. However, we cannot exclude the possibility that ADAM10 may be regulating the cleavage of N-cadherin as a downstream event following cleavage of the NOTCH1 receptor.

While this study establishes ADAM10 as indispensable for the early retinal development, the role of ADAM17 still remains unclear. Our results show that ADAM10 and ADAM17 exhibit an overlapping spatiotemporal expression pattern in the developing mouse retinae consistent with the expression of ADAM10 and ADAM17 previously reported for the developing chick retinae [34]. However, it should be pointed out that another study using a different antibody identified that in mice, the retinal expression of ADAM17 starts at P7 [55]. Furthermore, the same study revealed that in the adult (P175) mouse retinae ADAM17 expression is restricted to the ganglion cell layer, the inner and outer edge of the inner nuclear layer [55]. This discrepancy may have been caused by the variable specificity of the antibodies used as well as different time points selected for the analysis. Nonetheless, our findings unequivocally indicate that ADAM17 is dispensable for retinal development. In the developing chick retina, in addition to ADAM10, ADAM17 exhibits similar spatiotemporal expression pattern with ADAM9, ADAM12, ADAM13, ADAM22, and ADAM23 [34]. Except for ADAM9, that has been shown to have a role in the development of retinal vasculature and homeostasis [56, 57], the roles of other ADAM proteins during retinal development and homeostasis are unknown providing no information if ADAM17 may have functional overlaps with other ADAM proteins. Studies focusing on the role of ADAM17 as a regulator of retinal homeostasis are currently in progress.

## Supporting Information

S1 Fig Eye phenotypes of *Adam10* CKO and *Adam17* CKO mice. (TIF)

S2 Fig *Adam10* CKO eye phenotypes. (TIF)

S3 Fig BRN3B expression in E13.5 expression in E13.5 control and *Adam10* CKO retinae. (TIF)

S4 Fig Retinae of E13.5 N1-ICD and *Adam10* CKO mice. (TIF)

S1 Table A list of primers used in the study. (DOCX)
