# Supplementary figures and images for: A Disintegrin and Metalloproteinase10 (ADAM10) Regulates NOTCH Signaling during Early Retinal Development
Source: PLoS One. 2016 May 25;11(5):e0156184. doi: 10.1371/journal.pone.0156184 (PMC4880208; doi:10.1371/journal.pone.0156184)

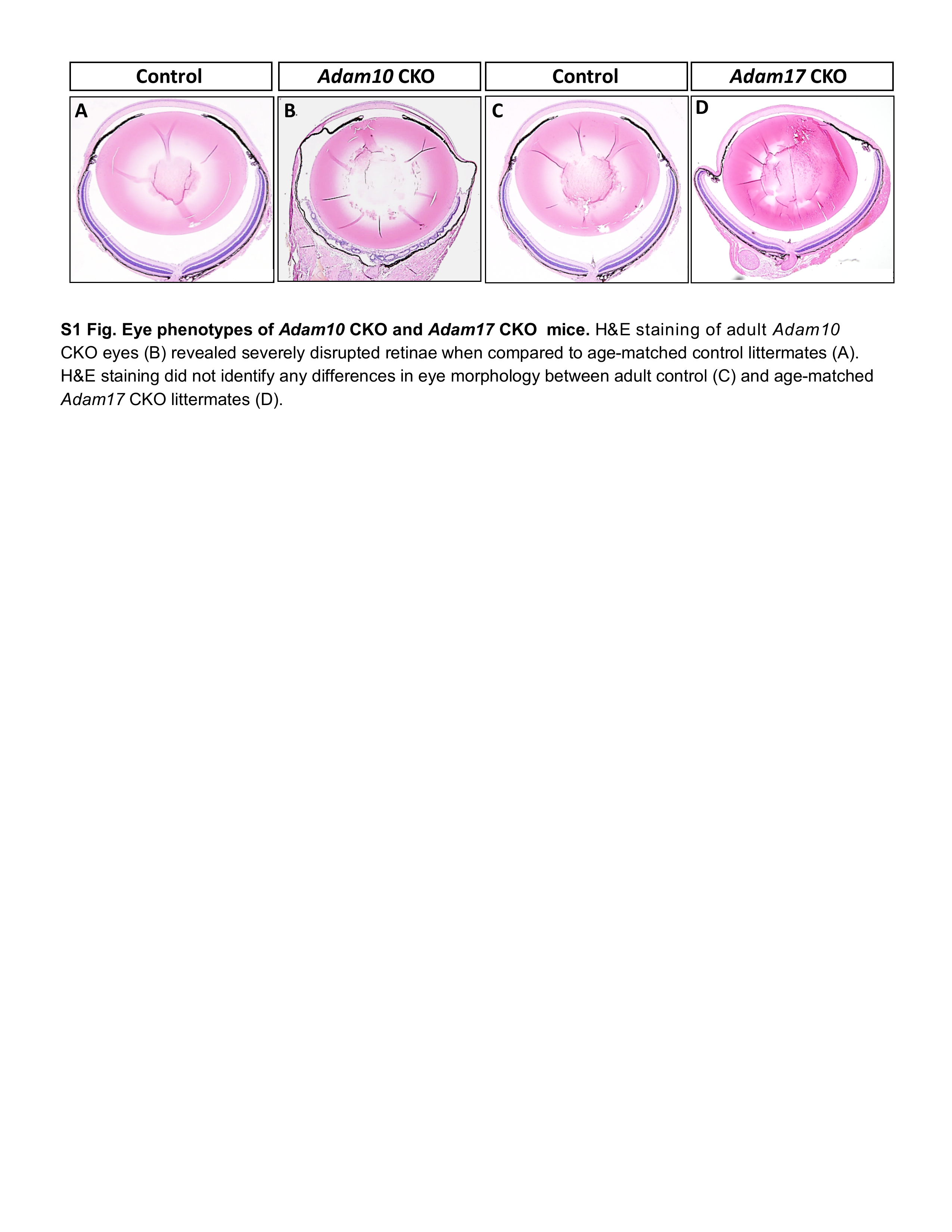

Supplement: S1 Fig — (TIF) [file pone.0156184.s001.tif]

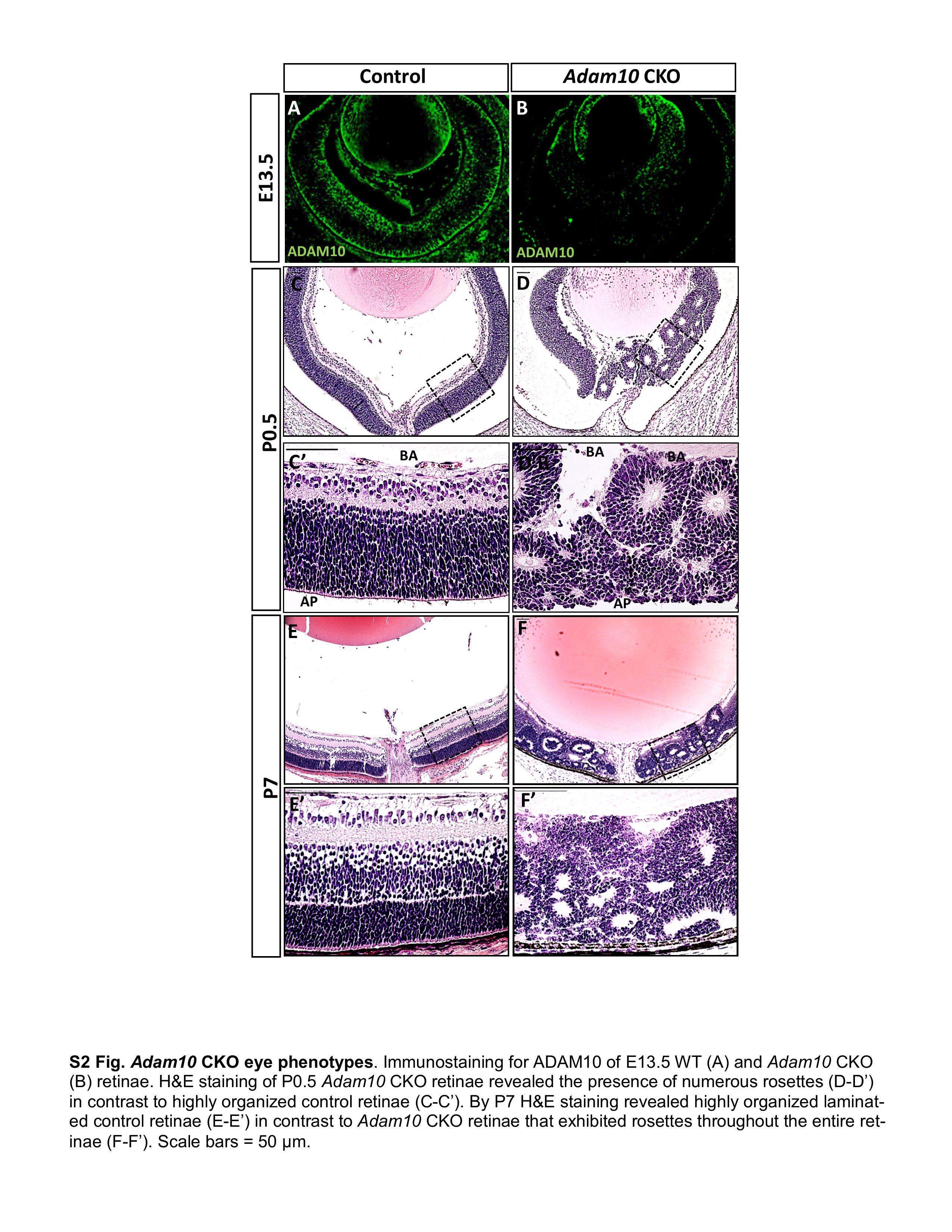

Supplement: S2 Fig — (TIF) [file pone.0156184.s002.tif]

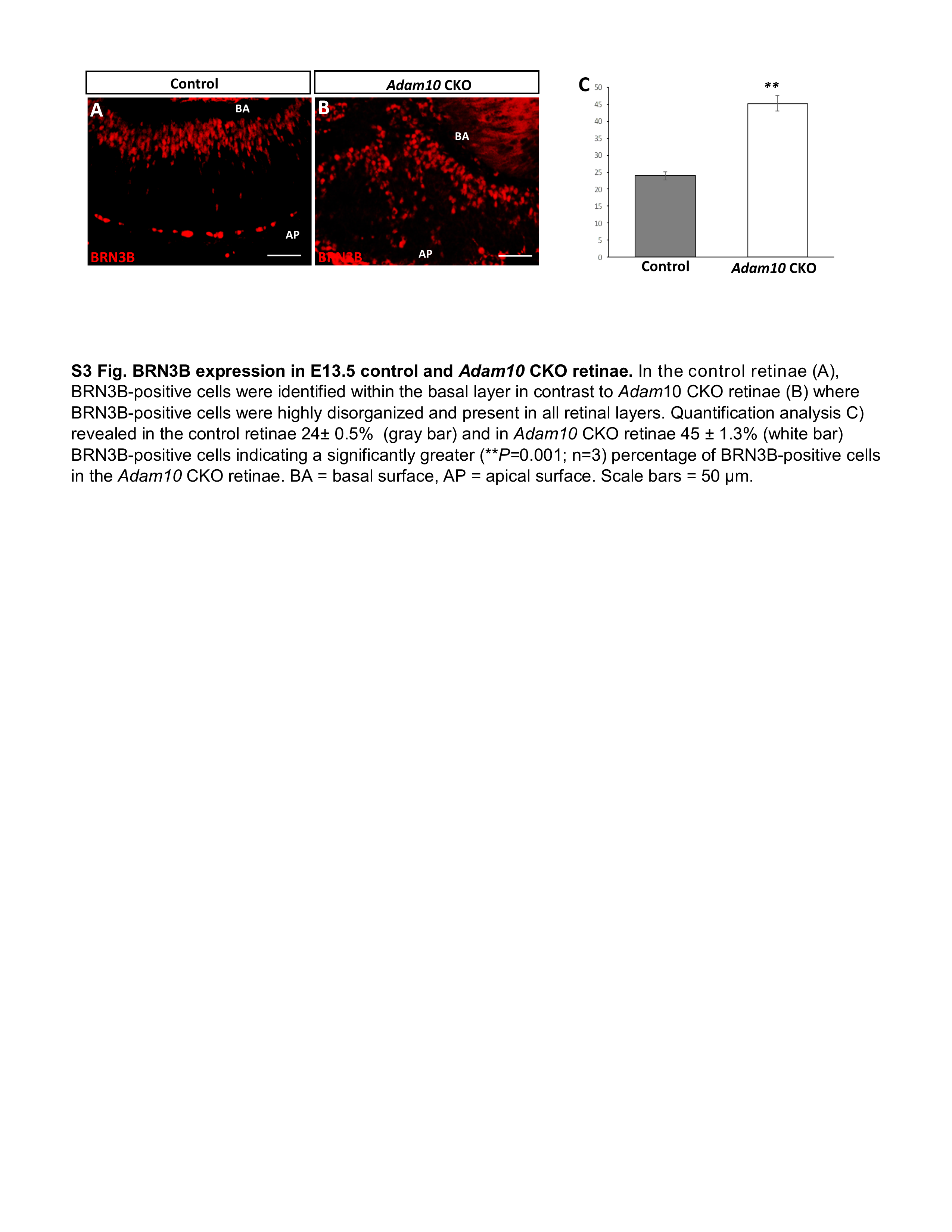

Supplement: S3 Fig — (TIF) [file pone.0156184.s003.tif]

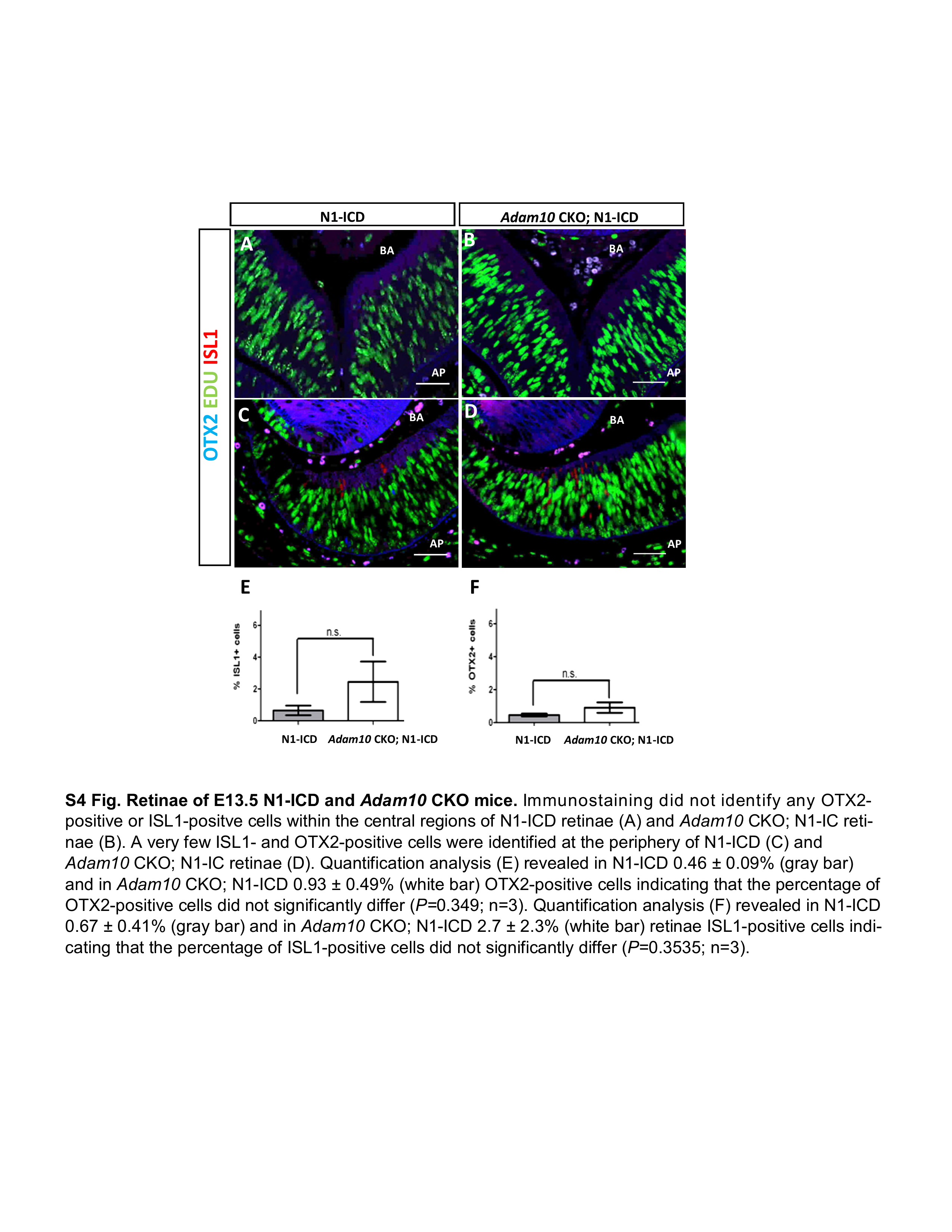

Supplement: S4 Fig — (TIF) [file pone.0156184.s004.tif]
