# Supplementary material for: A Disintegrin and Metalloproteinase10 (ADAM10) Regulates NOTCH Signaling during Early Retinal Development
Source: PLoS One. 2016 May 25;11(5):e0156184. doi: 10.1371/journal.pone.0156184 (PMC4880208; doi:10.1371/journal.pone.0156184)
Supplement: S1 Table — (DOCX) [file pone.0156184.s005.docx]

|  |  | **Table S1. A list of primers used in the study.** | | |
| --- | --- | --- | --- | --- |
| Primer | | | Sequence (5' ̶ 3') |  |
| Adam10F | | | GAGAGGAAAGAAAGTGGCAGA |  |
| Adam10R | | | AGTGGGTGGGTTAATGAGCA |  |
| Adam17 F | | | TCTGCGGAAGCTTTACTTTCCTG |  |
| Adam17 R | | | CAAAACACACAAACCACACATACTT |  |
| Cre F | | | TCGATGCAACGAGTGATGAG |  |
| Cre R | | | TTCGGCTATACGTAACAGGG |  |
| NICD-WT-F | | | CCAAAGTCGCTCTGAGTTGTTATC |  |
| NICD-WT-R | | | GAGCGGGAGAAATGGATATG |  |
| NICD-Mut-F | | | GAAAGACCGCGAAGAGTTTG |  |
| NICD-Mut-R | | | AAAGTCGCTCTGAGTTGTTAT |  |
| Adam10 sqRT-PCR-F | | | TGATACCTCTCATATTTACAC |  |
| Adam10 sqRT-PCR-R | | | AGAATTCAACTCCAGGAACT |  |
| Adam17 sqRT-PCR-F | | | CCATAAGGAAAAGGGATCTA |  |
| Adam17 sqRT-PCR-R | | | CCCAAGCATCCTTCTCTTCGTTT |  |
| Gapdh sqRT-PCR-F | | | CTTTGGCATTGTGGAAGGG |  |
| Gapdh sqRT-PCR-R | | | CCTCTCTTGCTGCAGTGTC |  |
